# Identification of novel pentose transporters in *Kluyveromyces marxianus* using a new screening platform

**DOI:** 10.1093/femsyr/foab026

**Published:** 2021-04-23

**Authors:** Lorena Donzella, Javier A Varela, Maria João Sousa, John P Morrissey

**Affiliations:** School of Microbiology, Environmental Research Institute, APC Microbiome Institute, University College Cork, Cork T12 K8AF, Ireland; Centre of Environmental and Molecular Biology, Department of Biology, University of Minho, *Campus* of Gualtar, R. da Universidade, Braga 4710-057, Portugal; School of Microbiology, Environmental Research Institute, APC Microbiome Institute, University College Cork, Cork T12 K8AF, Ireland; Centre of Environmental and Molecular Biology, Department of Biology, University of Minho, *Campus* of Gualtar, R. da Universidade, Braga 4710-057, Portugal; School of Microbiology, Environmental Research Institute, APC Microbiome Institute, University College Cork, Cork T12 K8AF, Ireland

**Keywords:** pentose, major facilitator superfamily, yeast, biotechnology, evolution, screen

## Abstract

The capacity of yeasts to assimilate xylose or arabinose is strongly dependent on plasma membrane transport proteins. Because pentoses comprise a substantial proportion of available sugars in lignocellulosic hydrolysates, their utilisation is centrally important for the development of second generation biorefineries. Relatively few native pentose transporters have been studied and there is intense interest in expanding the repertoire. To aid the identification of novel transporters, we developed a screening platform in the native pentose-utilising yeast *Kluyveromyces marxianus*. This involved the targeted deletion of twelve transporters of the major facilitator superfamily (MFS) and application of a synthetic biology pipeline for rapid testing of candidate pentose transporters. Using this *K. marxianus* ΔPT platform, we identified several *K. marxianus* putative xylose or arabinose transporter proteins that recovered a null strain's ability to growth on these pentoses. Four proteins of the HGT-family were able to support growth in media with high or low concentrations of either xylose or arabinose, while six HXT-like proteins displayed growth only at high xylose concentrations, indicating solely low affinity transport activity. The study offers new insights into the evolution of sugar transporters in yeast and expands the set of native pentose transporters for future functional and biotechnological studies.

## Teaser

New yeast screening platform to identify pentose transporters.

## INTRODUCTION

*Kluyveromyces marxianus* is now considered a credible alternative to *Saccharomyces cerevisiae* for specific biotechnological applications (Morrissey *et al*. 2015; Karim, Gerliani and Aïder 2020; Nurcholis *et al*. 2020; Rajkumar and Morrissey 2020). Intrinsic traits like rapid growth, thermotolerance, recognised safe status and capacity to grow on diverse sugars are important contributory factors. Its natural ability to assimilate pentoses is of particular interest because xylose and arabinose comprise up to 40% of available sugars in lignocellulosic biomass (LCB) (Takkellapati, Li and Gonzalez 2018). The future development of the circular bioeconomy requires the use of residual or dedicated biomass such as LCB, and viable biorefineries require the use of microbial cell factories that can efficiently use all available sugars in a feedstock. Although transport systems for glucose, lactose and galactose are well-described, there is limited knowledge on pentose transporters in *K. marxianus*.

It is known that monosaccharide uptake in yeasts mainly occurs through the HXT class of transporters, which are a subset of the major facilitator superfamily (MFS) (Leandro, Fonseca and Gonçalves 2009). Certain HXT proteins enable efficient import of hexoses, but their lower affinity for xylose and arabinose hinders pentose consumption in the presence of glucose and contributes to the diauxic growth observed on mixtures of these sugars (Young *et al*. 2014; Wang, Yu and Zhao 2016). The expansion of the *HXT* genes is well-studied in *S. cerevisiae* (Özcan and Johnston 1999), and previously we showed that these genes are also duplicated in *K. marxianus*, where they encode a mixture of high and low affinity glucose transporters (Varela *et al*. 2019a). Unlike *S. cerevisiae*, *Kluyveromyces* species also encode proteins in the HGT family of sugars transporters. As is the case with the *HXT* genes, the *HGT* genes are expanded in *K. marxianus*. The product of one of these genes, HGT1, has been extensively characterised in *Kluyveromyces lactis* as a high affinity glucose transporter, as well as a galactose transporter (Billard *et al*. 1996; Baruffini *et al*. 2006). Our prior work confirmed orthologous function for the first gene in the *K. marxianus HGT* cluster (Varela *et al*. 2019a), and other researchers demonstrated that the protein encoded by this gene was able to transport arabinose and xylose and thus named the gene *KmAxt1* (Knoshaug *et al*. 2015). Only HGT1/KmAxt1 has a known activity and previous work showed that other HGTs are either capable of low affinity galactose transport or have no known substrates (Varela *et al*. 2019a).

Because of the importance of pentose transport for biotechnology applications, we were interested in establishing whether any other of the HGT proteins, or indeed other putative transporters, were able to transport arabinose and/or xylose in *K. marxianus*. Platform strains unable to transport sugars are valuable tools to identify novel transporters and to study transport kinetics (Wieczorke *et al*. 1999; Alves-Araújo *et al*. 2005; Wijsman *et al*. 2019). Although it is possible to engineer *S. cerevisiae* to use pentoses quite efficiently, we reasoned that a platform in a yeast strain that could natively use pentose sugars would be a powerful resource that could also facilitate studies linking transport with metabolism and physiology. Accordingly, we constructed a *K. marxianus* strain deleted in 12 genes that was completely unable to grow on either xylose or arabinose. We designated this strain *K. marxianus* ΔPT and, following a screen of candidate transporters, we identified several novel xylose transporters, two of which are also involved in arabinose uptake. This work contributes to a better knowledge of sugar membrane transporters in *K. marxianus* and offers resources and tools to researchers interested in identifying, characterising and exploiting pentose transport proteins.

## MATERIALS AND METHODS

### Strains and growth conditions

*Kluyveromyces marxianus* strains used and constructed in this study are listed in Table 1. Strains were routinely cultured at 30°C in YP medium (10 g L^−1^ yeast extract, 20 g L^−1^ peptone) or minimal medium (MM) (5 g L^−1^ Urea, 3 g L^−1^ KH_2_PO_4_, 0.5 g L^−1^ MgSO_4_.7H_2_O, 100 μg mL^−1^ vitamin solution, 100 μg mL^−1^ trace elements solution), supplemented with 20 g L^−1^ of the appropriate sugar (D, Glucose; LAC, lactose). Composition of the vitamin and trace element solutions is reported in the Supplementary File 1. Where required for selection, 200 μg mL^−1^ of Hygromycin B (Sigma-Aldrich, St. Louis, MI, USA) was added to agar plates.

**Figure 4.**
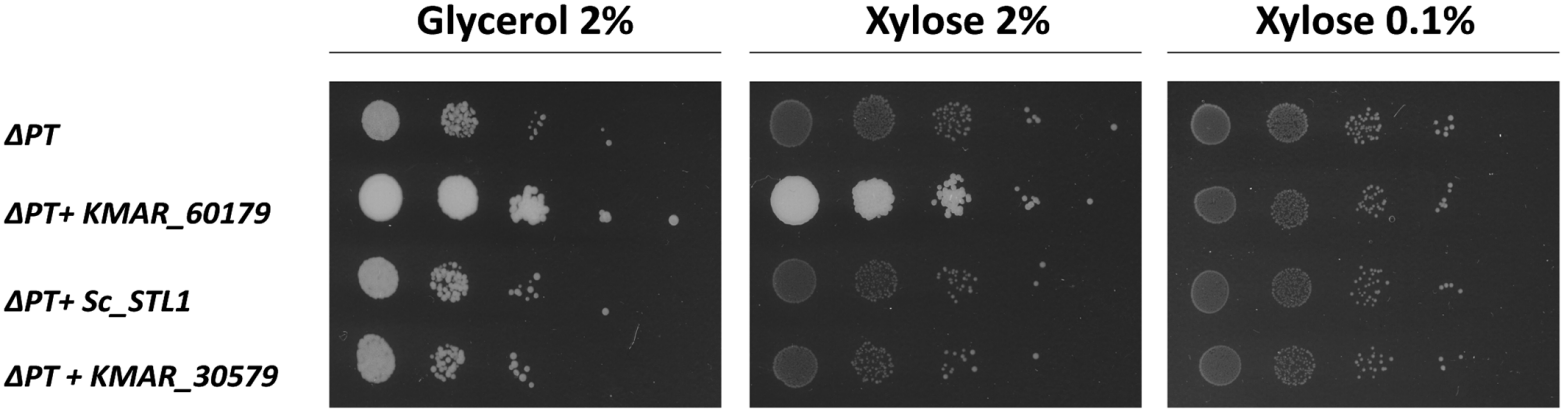
The *K. marxianus* STL1 protein, KMAR_60179, can mediate both glycerol and xylose transport. The two *K. marxianus* genes with homology to *S. cerevisiae STL1*, and *Sc_STL1*, were individually expressed on a plasmid in *K. marxianus* ΔPT. Following pre-growth on 2% lactose, strains were plated in serial dilution on agar plates containing 2% glycerol or 2% or 0.1% xylose as indicated. Plates were photographed after 96 h of growth at 30°C.

**Figure 5.**
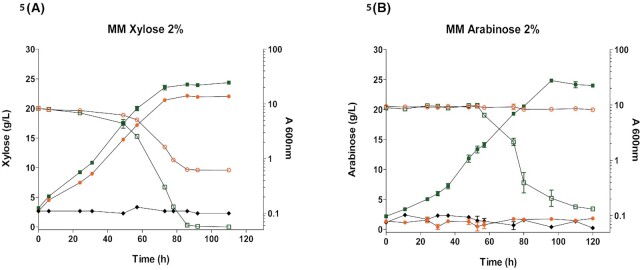
Application of *K. marxianus* ΔPT to compare the performance of putative low and high affinity pentose transporters. Genes encoding the candidate high affinity (KMAR_10531) and low affinity (KMAR_60179) pentose transporters were expressed from the *TEF1* promoter on the TU-DO-G418 plasmid in *K. marxianus* ΔPT. Following pre-growth on 2% lactose, the strains carrying *KMAR_10531* (green squares), *KMAR_60179* (orange circles), or empty plasmid (black diamonds) were inoculated in MM with 2% xylose **(A)** or arabinose **(B)** and grown at 30°C. Growth (solid symbols) and xylose or arabinose concentration (open symbols) were measured for 120 h.

**Table 1.** Strains used in this study.

Strain	Genotype	Parental strain	Mutation coordinates	Origins
*K. marxianus* NBRC1777	Wild-type strain			NITE Biological Research Centre, Japan
*K. marxianus Δhgt*	*Δkmar_10531, Δkmar_10530, Δkmar_10529, Δkmar_10528, Δkmar_10527*	*K. marxianus* NBRC1777	HGT: CHR I Δ1,131 464–1145439	Varela *et al*. 2019a
*K. marxianus Δkht*	*Δkmar_50347, Δkmar_50346, Δkmar_50345, Δkmar_50344, Δkmar_50343, Δkmar_50342*	*K. marxianus* NBRC1777	KHT: CHR V Δ 1251–4853, Δ 4926–16 261, Δ 16 417–20938	This study
*K. marxianus ΔhgtΔkht*	*Δhgt Δkht*	*K. marxianus Δkht*	HGT: CHR I Δ1,131 464–1145439 KHT: CHR V Δ 1251–4853, Δ 4926–16 261, Δ 16 417–20938	This study
*K. marxianus* ΔPT	*Δhgt Δkht Δkmar_60179*	*K. marxianus Δhgt Δkht*	HGT: CHR I Δ1,131 464–1145439 KHT: CHR V Δ 1251–4853, Δ 4926–16 261, Δ 16 417–20938 60179: CHR VI Δ 134–1710	This study
*S. cerevisiae CEN.PK 113–7D*	Wild-type strain			Nijkamp *et al*. 2012

Serial dilutions on 2% agar plates were used to determine the capacity of wild-type and engineered strains to use specific sugars. For these experiments, a single colony was inoculated into 5 mL YPLAC in a 15 mL Sterilin tube and this was incubated overnight at 30°C. Cells were then harvested by centrifugation, washed with water, and a serial dilution was prepared in water. About 5 μL of each dilution were spotted onto minimal media (MM) plates containing D-Glucose, α-Lactose, D-Xylose, L-Arabinose or glycerol to a final concentration of 20 or 1 g L^−1^_._ Plates were incubated for up to 120 h at 30°C and the growth was assessed. To validate the results of the plate assays by testing growth in liquid, a single colony of each engineered strain was inoculated into 10 mL MM media (2% xylose or 2% arabinose) in a 100 mL flask and incubated for 96 h at 30°C. Cells were harvested by centrifugation, the supernatant was used for sugar consumption analysis with high-performance liquid chromatography (HPLC) and the pellet was used to determine dry cell weight DCW, expressed as g L^−1^.

Growth curves on xylose were performed in 500 mL shake flasks as follows. Overnight inoculum in YPLAC was prepared as for the serial dilution experiments and 100 mL of 20 g L^−1^ xylose MM medium was inoculated to a starting A_600nm_ of 0.1. The flasks were incubated at 30°C on an orbital shaker at 200 rpm and A_600nm_ was measured every 6–12 h for 160 h. Consumption of xylose was determined by measuring the sugar remaining in the medium after various periods of growth. About 1.5 mL of culture were harvested and spun down to prepare supernatant for HPLC. For the analysis, 800 μL of supernatant were injected into a Hewlett-Packard (HP) 1090 instrument using a Bio-Rad HPX-87H hydrogen ion resin column and an HP 1047A external refractive index detector. The mobile phase, 0.001 N H_2_SO_4_, was run at 55°C at a flow rate of 0.6 ml min^−1^. Standards were used quantify sugars. *E. coli* DH5α was used for cloning purposes. The strain was grown in LB medium (5 g L^−1^ yeast extract, 10 g L^−1^ bactopeptone, 10 g L^−1^ NaCl) or LB agar plates supplemented with 100 μg mL^−1^ ampicillin.

### *In silico* identification of Potential Pentose Sugar Transporters

Potential sugar porters were identified by the combination of different bioinformatic approaches. The predicted proteome of *K. marxianus* NBRC 1777 was retrieved from the NCBI Assembly platform (https://www.ncbi.nlm.nih.gov/) and then compared against the TransportDB 2.0 database to identify possible sugar transporters (Elbourne *et al*. 2017). After filtering out hits with low identity (<35%) and coverage (<80%), the resulting sequences were submitted to the TMHMM server for the prediction of transmembrane domains. All proteins predicted to contain 11 or more transmembrane domains were considered as potential sugar transporters. Sequences belonging to the MFS superfamily were extracted from this list and the 27 predicted proteins remaining further analysed to try to predict function. Synteny and evolutionary conservation were inspected using the Yeast Gene Browser (YGOB, www.ygob.ucd.ie) (Byrne and Wolfe 2005). Homologous sequences from *S. cerevisiae* and *K. lactis* were recovered and sequence alignment was performed using the MUSCLE algorithm 3.8 (Edgar *et al*. 2004) and visualized using Jalview v.2 (Waterhouse *et al*. 2009). Maximum likelihood phylogenetic analysis with 600 times bootstrap and tree building of the putative sugar transporters compared with known xylose transporters were performed using MEGA6/MUSCLE (Tamura *et al*. 2013). Preliminary 3D-studies were performed to compare the binding site of KMAR_60179 transporter from *K. marxianus* to the known glycerol transporter Sc_STL1 from *S. cerevisiae* in order to evaluate the capability of transport of xylose. Protein structure prediction was performed using the ITASSER platform (https://zhanglab.ccmb.med.umich.edu/I-TASSER/) (Yang and Zhang 2015) and the structures were visualized and investigated with PyMOL v.2.3 (Delano 2002). Three different naming conventions have been used for *K. marxianus* genes, depending on the strain annotated. Since our study uses strain NBRC 1777, we retained the designations from this annotation. To enable comparison to previous studies, however, we include a table showing the equivalence of the different transporters with which we worked (Table S1, Supporting Information).

### Construction of *K. marxianus* mutants

For general screens of gene function, we used the CRISPR-Cas9 system previously developed in our group (Juergens *et al*.^2018^; Varela *et al*. 2019a) to generate inactivating mutations by non-homologous end-joining (NHEJ) in *K. marxianus* NBRC 1777. This uses pUCC001 (all plasmids listed in Table 2), which contains the Cas9 gene and the elements required to express the guide RNA (gRNA) molecule for targeted double stranded breaks. The design of the gRNA sequences specific to target genes was performed using the software sgRNAcas9 (Xie *et al*. 2014) and CRISPRdirect (Naito *et al*. 2015). For cloning into pUCC001, 20nt target and its reverse complement primers were ordered with suitable overhangs for cloning via Golden Gate Assembly (New England Biolabs, Inc., Ipswich, MA, United States). The presence of the gRNA target sequences in the pUCC001 plasmid was confirmed by colony PCR using the forward target primer and BSA-R. All primers used in this study were ordered from Sigma-Aldrich^®^ and are listed in Table S2 (Supporting Information). Following cloning, the disruption plasmids were transformed into *K. marxianus* NBRC1777 using a variation of the LiAc/SS carrier DNA/PEG method (Gietz and Schiestl 2007*)*. Briefly, an overnight yeast culture in YPLAC were used to inoculate a shake flask containing YPLAC medium at an initial A_600nm_ of 0.5. Cultures were then incubated at 30°C until an A_600nm_ of 2.0 was reached, then harvested and transformed with 300 ng of plasmid DNA. After heat shock, cells were recovered in 1 mL YPLAC medium for 3 h and plated on selective plates containing hygromycin B 100 μg mL^−1^. Colonies were recovered and restreaked on selective medium (hygromycin B) to confirm presence of the plasmid. Colony PCR was carried out using diagnostic primers (diag. F and R) for each gene, and PCR products were then sequenced to determine whether an inactivating mutation had been introduced during repair of the dsDNA break. To allow loss of the pUCC001 plasmid from mutants, strains were grown on YPLAC broth overnight without selection and diluted 1/10 into fresh YPLAC next morning. This was repeated twice, and then single colonies were tested to confirm that they were Hygromycin B sensitive and hence had lost the plasmid. This allowed sequential construction of mutants where required. All sequencing was carried out by GATC Services—LightRun Tube Eurofins Genomics.

**Table 2.** CRISPR-Cas9 and complementation plasmids used in this study. Restriction enzyme sites are reported in superscript and sgRNA target are indicated in bold.

CRISPR-Cas9 Plasmids	Relevant characteristics	Source
pUDP002	ori ampR panARS(OPT) AgTEF1p-hph-AgTEF1t ScTDH3pBsaI BsaIScCYC1t AaTEF1p-Spcas9D147Y P411T-ScPHO5t	Juergens *et al*. 2018
pUCC001	[pUDP002] HH-BsaI BsaI-gRNAscaffold-HDV	Rajkumar *et al*. 2019
pUCC001- HGT	[pUCC001] sgRNA-HGT	Varela *et al*. 2019a
pUCC001- KHT	[pUCC001] sgRNA-KHT	This study
pUCC001- 20551	[pUCC001] sgRNA-KMAR_20551	This study
pUCC001- 30579	[pUCC001] sgRNA-KMAR_30579	This study
pUCC001- 50027	[pUCC001] sgRNA-KMAR_50027	This study
pUCC001- 60179	[pUCC001] sgRNA-KMAR_60179	This study
**Complementation plasmids**	**Relevant characteristics**	**Source**
pYTK001	Storage vector for cloning gene parts	Lee *et al*. 2015
pYTK_CYC1	[pYTK001] CYC1 TT	
pYTK013	[pYTK001] pTEF1	Rajkumar *et al*. 2019
p426	ori ampR URA3 pTEF1- HGT/KHT—CYC1 TT	Varela *et al*. 2019a
p426-E03650	[p426]-TEF1-E03650	Varela *et al*. 2019a
p426-E03670	[p426]-TEF1-E03670	Varela *et al*. 2019a
p426–50345	[p426]-TEF1–50345	Varela *et al*. 2019a
p426–50344	[p426]-TEF1–50344	Varela *et al*. 2019a
p426–50343	[p426]-TEF1–50343	Varela *et al*. 2019a
p426-A20920	[p426]-TEF1-A02920	Varela *et al*. 2019a
p426-A02930	[p426]-TEF1-A02930	Varela *et al*. 2019a
p426-A02950	[p426]-TEF1-A02950	Varela *et al*. 2019a
p426-A02960	[p426]-TEF1-A02960	Varela *et al*. 2019a
pTU-DO-G418	ampR KmARS/CEN7 KanMX BsaI sfGFP BsaI	Rajkumar and Morrissey 2020
pTU-DO-G418- 50342	pTEF1-KMAR_50342-CYC1 TT	This study
pTU-DO-G418- 50343	pTEF1-KMAR_50343-CYC1 TT	This study
pTU-DO-G418- 50344	pTEF1-KMAR_50344-CYC1 TT	This study
pTU-DO-G418- 50345	pTEF1-KMAR_50345-CYC1 TT	This study
pTU-DO-G418- 50346	pTEF1-KMAR_50346-CYC1 TT	This study
pTU-DO-G418- 10527	pTEF1-KMAR_10527-CYC1 TT	This study
pTU-DO-G418- 10528	pTEF1-KMAR_10528-CYC1 TT	This study
pTU-DO-G418- 10529	pTEF1-KMAR_10529-CYC1 TT	This study
pTU-DO-G418- 10530	pTEF1-KMAR_10530-CYC1 TT	This study
pTU-DO-G418- 10531	pTEF1-KMAR_10531-CYC1 TT	This study
pTU-DO-G418- 50027	pTEF1-KMAR_50027-CYC1 TT	This study
pTU-DO-G418- 60179	pTEF1-KMAR_60179-CYC1 TT	This study
pTU-DO-G418- 30579	pTEF1-KMAR_30579-CYC1 TT	This study
pTU-DO-G418- Sc_STL1	pTEF1-Sc_STL1-CYC1 TT	This study
pTU-DO-G418- Sc_GAL2	pTEF1-Sc_GAL2-CYC1 TT	This study

A variation of the aforementioned CRISPRCas9 system based on homology dependent repair (HDR) was used for construction of stable mutants that did not have the potential to revert. Previously, we showed that it was possible to inactive the entire *HGT* gene cluster using a single gRNA that targeted a common sequence present in all *HGT* genes generating strain *Δhgt* (Varela *et al*. 2019a). This deletion arises due to recombination between the cut first and last genes in the cluster and the same strategy was used to inactive the entire *KHT/HXT* locus generating strains *Δkht and ΔhgtΔkht* (Fig. S1, Supporting Information). For disruption of *KMAR_60179*, a repair fragment was constructed by designing primers RF_60179 F and RF_60179 R, which shared a 30bp overlap and had 80b homology to the 5′ and 3′ regions flanking *KMAR_60179*, respectively. The 5′ and 3′ homology arms were annealed and extended by overlap extension PCR using Q5® High-Fidelity DNA Polymerase (New England Biolabs, Ipswich, MA). Successful construction of the final repair fragment of 190 bp was confirmed by gel electrophoresis and 1 μg repair fragment was then co-transformed with 200 ng pUCC001–60179 into *K. marxianus ΔhgtΔkht* to generate *K. marxianus ΔhgtΔkhtΔkmar_60179* (later named *K. marxianus* ΔPT). All deletion mutants were confirmed by diagnostic colony PCR with sequencing across the deletion (Fig. S2, Supporting Information). The precise co-ordinates of deletions are listed in Table 1, using the genomic co-ordinates from the NBRC1777 genome sequence (Inokuma *et al*. 2015).

### Construction of plasmids expressing candidate pentose transporters and complementation assays

For expression studies, the *KHT* and *HGT* genes and *KMAR_60179* were cloned into the base plasmid pTU-DO-G418 using a Golden Gate assembly as shown in (Fig. S3, Supporting Information). pTU-DO-G418 is similar to pMTU-DO-G418 but carries an ampicillin rather than a kanamycin resistance gene (Rajkumar and Morrissey 2020). We use the YTK standard in *K. marxianus* as previously reported by Lee *et al*. 2015 and Rajkumar *et al*. 2019. The strategy entailed *in vitro* assembly of the *S. cerevisiae TEF1* promoter (p*TEF1*), the open reading frame of the transporter, the *S. cerevisiae CYC1* transcriptional terminator (*CYC1* TT) and the plasmid backbone. The p*TEF1* part was already available in the plasmid, YTK013, and a similar storage plasmid with *CYC1* TT was made by amplifying the part from *S. cerevisiae* CENPK 113–7D with primers ScCYC1 F-R and cloning into the storage plasmid YTK001 to create plasmid YTK_CYC1. For construction of the expression vectors for *KMAR_60179, KMAR_10529, KMAR_50027, KMAR_50346, KMAR_30579*, *ScSTL1 and ScGAL2*, the open reading frames were amplified by PCR with primers containing overlapping regions for *pTEF1* and the *CYC1 TT* and the vector assembled as previously described (Rajkumar *et al*. 2019). *KMAR_10527, KMAR_10528, KMAR_10530, KMAR_10531, KMAR_50342, KMAR_50343, KMAR_50344, KMAR_50345* had already been cloned between pTEF1 and CYC TT in the p426 plasmid series (Varela *et al*. 2019a), and, in this case, the entire cassette promoter-gene-terminator was amplified from the p426 plasmid with primers ScTEF1-F and ScCYC1_R and cloned into pTU-DO-G418 by Golden Gate assembly, as described above. All the PCR amplifications were performed using Q5 High-Fidelity Master Mix (New England Biolabs). The resulting plasmids were introduced in competent *E. coli* DH5α cells (Invitrogen 11 319–019) by heat shock and the cells were plated on LB plates with ampicillin 100 μg mL^−1^. After 24 h, white colonies were picked and screened by colony PCR with K1F and BsmbI_TUDOg418_R primers and plasmids were extracted from the positive colonies and sequenced to check for correct assembling. *Kluyveromyces marxianus* strains were transformed as described earlier but with selection for G418 resistance. The plasmids used for the expression of candidate transporters are reported in Table 2.

## RESULTS

### Identification of putative sugar transporters in *Kluyveromyces marxianus*

**Figure 1.**
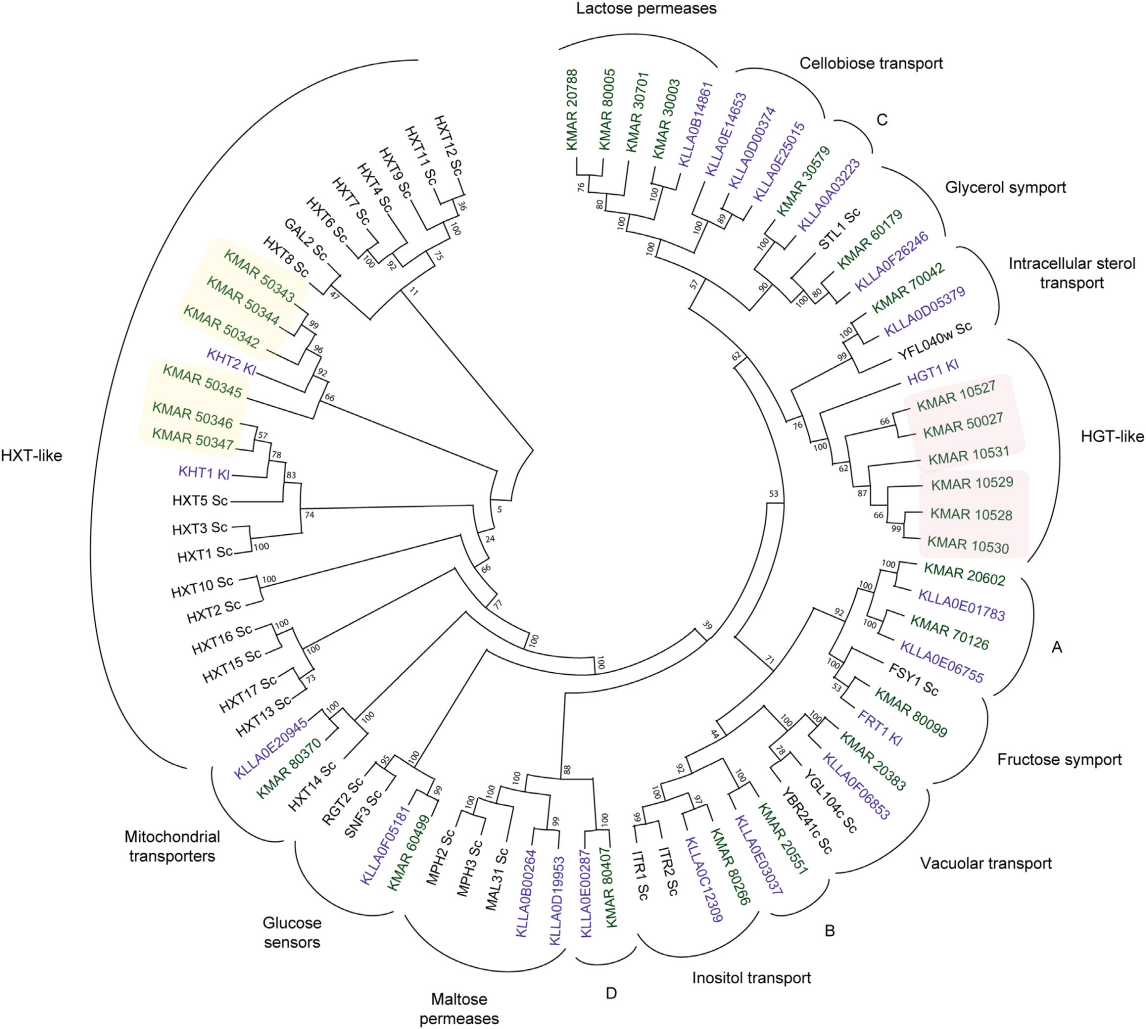
Phylogenetic comparison of potential MFS transporters between *K. marxianus*, *K. lactis* and *S. cerevisiae*. Bioinformatic analysis identified 27 predicted proteins in *K. marxianus* NBRC 1777 that satisfied the criteria of being potential sugar transporters (MFS family with 11 or 12 transmembrane domains) (in green). Homologous sequences in *S. cerevisiae* and *K. lactis* (in black and purple, respectively) were retrieved and phylogenetic analysis carried out using MEGA6/MUSCLE. The tree is derived from maximum-likelihood analysis with 600 times bootstrapping. The expanded KHT/HXT and HGT families in *K. marxianus* are highlighted with yellow and pink squares, respectively. Labelling is used to indicate the known or predicted function of proteins where data are available. The labels A—D mark *Kluyveromyces* proteins of unknown function that lack clear orthologues in *S. cerevisiae*. Sc_Fsy1 is a fructose transporter that was acquired by horizontal gene transfer in wine strains and is not a native *S. cerevisiae* protein.

A bioinformatic approach was taken to identify all potential sugar transporters in *K. marxianus*. To do this, the predicted proteome of *K. marxianus* NBRC1777 was compared to the TransportDB 2.0 database to identify candidate MFS transporters. These were then screened on the TMHMM server to select proteins with at least 11 transmembrane domains. This analysis yielded 27 predicted proteins of the Major Facilitator Superfamily (MFS). These 27 proteins were then compared to homologous proteins from *K. lactis* and *S. cerevisiae* to establish gene orthology (Fig. 1). The majority of the proteins were distributed in clusters that include at least one characterised protein from *S. cerevisiae* and therefore function can be inferred. There are, however, a number of clusters containing proteins present in one or more *Kluyveromyces* species that are absent from *S. cerevisiae*. First, both *Kluyveromyces* species encode the lactose transporter Lac12, though this gene has been amplified to four copies in *K. marxianus* (*KMAR_20788, KMAR_80005, KMAR_30701, KMAR_30003*). Second, *K. lactis* encodes three copies of a dedicated cellobiose transporter (*KLLA0E14653, KLLA0D00374, KLLA0E25015)* and, as reported, this gene is absent in *K. marxianus* (Varela *et al*. 2019b). Third, both species encode HGT1 for high affinity glucose transport, though, again as reported, this gene family is expanded in *K. marxianus* where 5 tandem copies are present on chromosome 1 and a further single homologue on chromosome 5. Fourth, both *Kluyveromyces* species encode the fructose transporter Fsy1 that is absent in *S. cerevisiae*, as well as two uncharacterised proteins of related sequence (marked ‘A’ in Fig. 1). *FSY1* is known to have been acquired by horizontal gene transfer in wine strains and is not a native *S. cerevisiae* gene (Galeote *et al*. 2010). The maltose transporter cluster is present in both *S. cerevisiae* and *K. lactis* but is absent in *K. marxianus*, consistent with the species’ maltose negative phenotype. Interestingly, *Kluyveromyces* yeasts encode a deeper branching transporter of unknown function that is related to maltose transporters (marked D in Fig. 1). In a similar fashion, there are deeper branching *Kluyveromyces*-specific proteins that are related to the inositol transporter Itr1/2 and the glycerol transporter STL1 (marked B and C, respectively, in Fig. 1). Substantial differences between the two *Kluyveromyces* species and *S. cerevisiae*, and between *K. lactis* and *K. marxianus* are evident when examining candidate transporters in the HXT

-like family of proteins. Orthologues of the *S. cerevisiae* high and low affinity HXT glucose transporters are present in *Kluyveromyces* and the expansion of these in *K. marxianus* relative to *K. lactis* was previously described (Varela *et al*. 2019a). The *K. lactis HXT* homologues were originally named *KHT1* (*RAG1*) and *KHT2*, thus we retain the *KHT* nomenclature in this study, though the ancestral relationship is clear. Thus, *K. marxianus* NBRC1777 has two proteins related to the low affinity glucose transporter KHT1 (Rag1) and four proteins related to the high affinity glucose transporter KHT2. These are in tandem repeats present in syntenic location with the ancestral *S. cerevisiae HXT* locus (*HXT4–HXT 1–HXT5/HXT7–HXT6–HXT3*). *Kluyveromyces* also have a HXT14 orthologue but lack orthologues of other HXT-like proteins such as the galactose transporter, GAL2, or the polyol transporters HXT15/16 and HXT13/17 (Jordan *et al*. 2016).

*Saccharomyces cerevisiae* is unable to efficiently transport pentose sugars, and since we were especially interested in identifying transporters of xylose and/or arabinose, we also performed a phylogenetic analysis comparing the 27 *K. marxianus* MFS proteins to known pentose transport*ers of Scheffersomyces stipitis, Debaryomyces hansenii, Meyerozyma guilliermondii (*syn*. Pichia guilliermondii)* and *Penicillium chrysogenum* (Fig. S4, *Supporting Information*). The *M. guilliermondii and P. chrysogenum* proteins cluster with the *Kluyveromyces* HGT-like family whereas the *Scheffersomyces* and *Debaryomyces* proteins form a separate unrelated cluster. In this regard, it is notable that the only *K. marxianus* protein that was known at this time to transport pentoses, KmAxt1, is encoded by a *HGT*-like gene, *KMAR_10527* (Knoshaug *et al*. 2015) whereas the most efficient *S. cerevisiae* protein that can transport xylose is an engineered derivative of the HXT family protein, GAL2 N376F (Farwick *et al*. 2014). Since both of these gene families are expanded in *K. marxianus*, we considered it possible that additional pentose transporters might be found in either of the gene clusters.

### Construction of a platform *K. marxianus* strain unable to grown on pentose sugars

**Figure 2.**
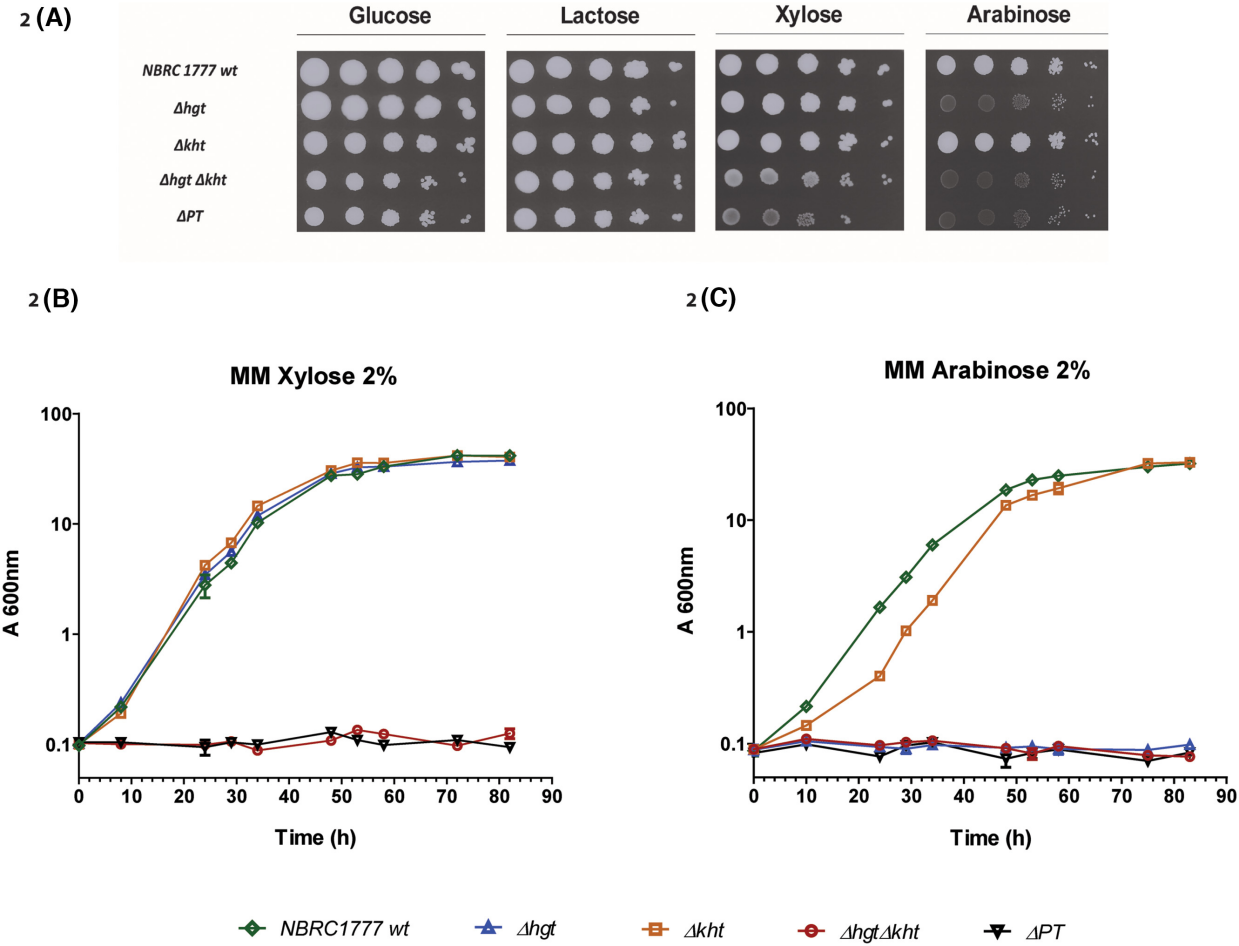
The *K. marxianus* platform strain ΔPT is unable to grow on the pentose sugars, xylose and arabinose. **A**. Growth of mutants on MM containing various sugars (2%). The ∆*hgt* mutant failed to grow on arabinose, indicating that all the functional arabinose transporters are encoded within the *HGT* cluster. The double mutant ∆*hgt*∆*kh*t grew slowly on xylose indicating both the presence of at least one xylose transporter in the KHT/HXT cluster and at least one other xylose transporter elsewhere in the genome. The ΔPT strain, which also deleted *KMAR_60179* in the ∆*hgt*∆*kht* background, failed to grow on either xylose or arabinose indicating the inactivation of all functional pentose transporters. **B-C***K. marxianus* NBRC 1777 wild-type (green diamonds), ∆*hgt* (blue triangles), ∆*kht* (orange squares), ∆*hgt*∆*kht* (red circles) and ΔPT (black triangles) were pre-grown on lactose medium and inoculated into 2% xylose or arabinose and cultivated in flasks at 30°C for 96h.

We decided to generate a *K. marxianus* pentose-negative strain to enable us to express individual transporters to establish if additional pentose transporters could be found. In a previous study, we used CRISPRCas9 to delete the five tandem *HGT* genes and here we employed the same method to delete the entire cluster of *KHT*-like genes in a single step. For this, we designed a CRISPR sgRNA to cut within a conserved region of the *KHT* genes. Following transformation with a plasmid expressing this sgRNA and Cas9, we were able to recover transformants in which all six genes were deleted due to a recombination between the first and the last gene, which generated a non-functional gene (Fig. S1, Supporting Information). The same method was used in both the wild-type and the Δ*hgt* strain, thus we had three strains Δ*hgt*, Δ*kht*, and Δ*hgt* Δ*kht*. We tested the capacity of these mutants to grow on lactose (positive control condition), glucose, xylose and arabinose (Fig. 2A). The Δ*hgt* strain failed to grow on arabinose but showed no growth defects on glucose or xylose. The Δ*kht* mutant did not show any phenotype but Δ*hgt*Δ*kht* was mildly impaired for growth on glucose, failed to grow on arabinose, and grew more slowly on xylose. The slower growth of *ΔhgtΔkht* was more apparent at earlier timepoints (Fig. S8, Supporting Information) but the timepoint of 96h (on xylose and arabinose) was chosen here to detect the influence of transporters that might have delayed or low expression. These data indicate that all functional arabinose transporters are encoded within the *HGT* cluster, that proteins capable of transporting xylose are found in both the *HGT* and *KHT* clusters, and that at least one other xylose transporter is encoded elsewhere in the genome.

In an effort to identify this additional xylose transporter, we used CRISPR-mediated NHEJ to systematically inactivate other genes that had been identified in the bioinformatic analysis in the Δ*hgt*Δ*kht* strain (Fig. S4, Supporting Information) as well as in the parental wild-type (Fig. S8, Supporting Information). Mutation of *KMAR_20551*, *KMAR_50027* or *KMAR_30579* did not show a phenotype but inactivation of *KMAR_60179* in the Δ*hgt*Δ*kht* background yielded a strain unable to grow on xylose. For subsequent analysis, we wanted to be sure that the mutant could not revert, so we generated a precise deletion of *KMAR_60179* in the Δ*hgt*Δ*kht* background using a CRISPR homology dependent repair (HDR) method and named this pentose transporter-deficient strain *K. marxianus* ΔPT as it failed to grow on pentose sugars (Fig. 2A). As agar plate assays often show slight background growth, we also carried out a liquid growth assay on 2% xylose (Fig. 2B) or 2% arabinose (Fig. 2C), which showed a complete absence of growth for the ΔPT strain, confirming its suitability as a screening host. Interestingly, *ΔhgtΔkht* also failed to grow, which is consistent with an idea that the slower growth seen on plates for this strain (Fig. 2A) is due to a transporter that may have either low activity or a low level of expression. Alternatively, it may be that, under the growth conditions of this experiment, the gene is expressed on solid but not liquid medium.

### Characterization of new putative pentose transporters

**Figure 3.**
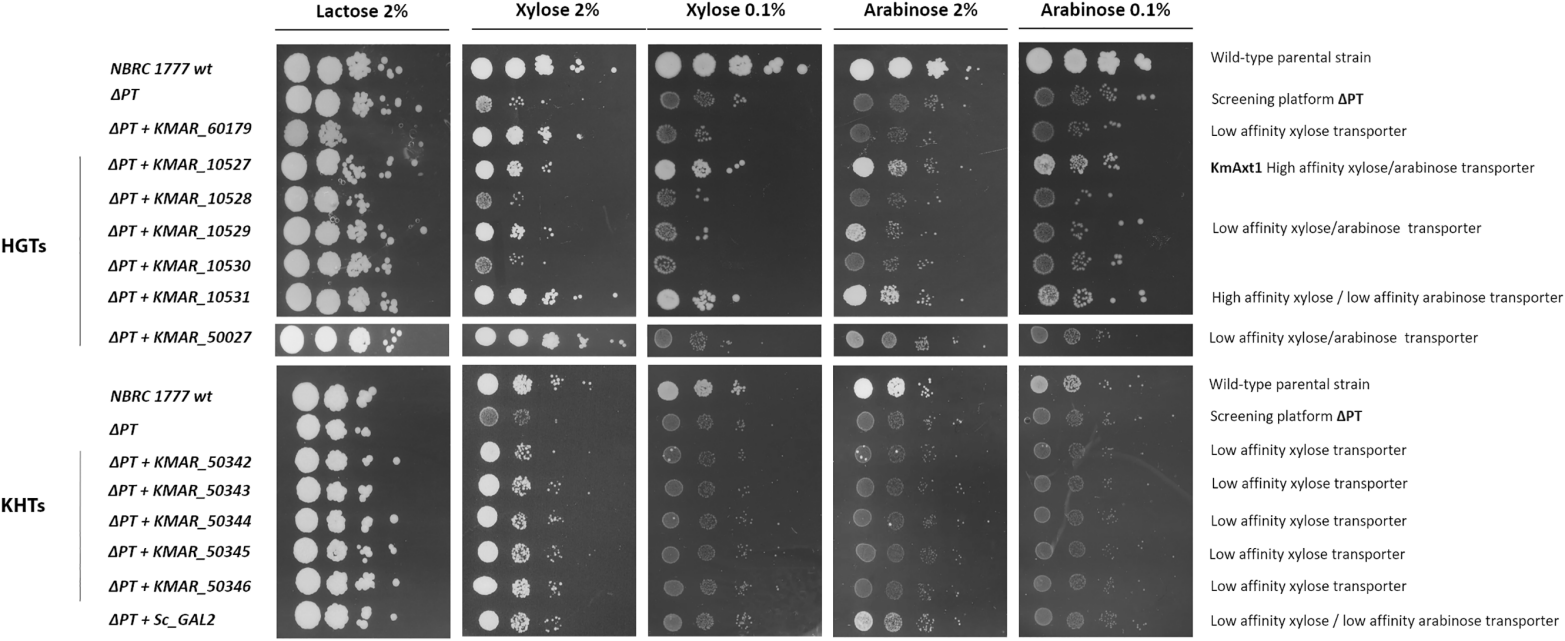
Identification of pentose transporters in *K. marxianus* ∆PT. Candidate native *K. marxianus* pentose transporters were individually expressed on a plasmid in the screening platform *K. marxianus* ΔPT. Strains were pre-grown on 2% lactose and then plated in serial dilution on agar plates containing either 2% or 0.1% xylose or arabinose as indicated. Plates were photographed after 96h of growth at 30°C.

The pentose-negative strain ΔPT was then used to establish which of the *HGT* and *KHT* genes encoded functional pentose transporters. For this, we individually cloned candidate genes into a plasmid where expression was driven from the constitutive Sc*TEF1* promoter. These plasmids were transformed into *K. marxianus* ΔPT, and strains expressing each of the thirteen genes were plated on medium containing xylose or arabinose at 2% or 0.1% concentration (Fig. 3). The two sugar concentrations were used to distinguish potential high and low affinity transporters, as low affinity transporters would only support growth at the higher sugar concentration. Subsequently, all the stains were tested in a liquid assay where biomass (cell dry weight) and % sugar consumed after 96 h of growth was determined (Table 3). Two of the HGT-like proteins, KMAR_10527 (KmAxt1) and KMAR_10531 supported growth at 0.1% and 2% concentrations of both sugars on plates and in liquid they enabled consumption of > 90% xylose and 45%–66% arabinose. Two other HGT-like proteins, KMAR_10529 and KMAR_50027, only allowed growth on these sugars at 2% on plates and led to consumption of 66%–59% xylose and 42%–59% arabinose. The remaining two HGT-like proteins, KMAR_10528 and KMAR_10530 transport neither sugar on plates nor in liquid growth. These data are consistent with a conclusion that KMAR_10527 (KmAxt1) and KMAR_10531 are high affinity and KMAR_10529 and KMAR_50027 low affinity pentose transporters. The ∆PT strains expressing the *KHT* genes reintroduced individually displayed only growth at 2% xylose on plates and showed xylose consumption ranging from 55%–85% in liquid. A similar phenotype was seen for a strain expressing *KMAR_60179*, indicating the five KHT proteins and KMAR_60179 have characteristics of a low-affinity xylose transporter. For reasons unexplained, we were unsuccessful in our efforts to clone the sixth KHT-encoding gene *KMAR_50347*. Based on the sequence similarity to KMAR_50346 (Fig. 1), however, it is likely that KMAR_50347 is functionally similar to the other KHT proteins with regard to pentose transport. The identification of KMAR_60179 as a xylose transporter was unexpected since this is a homologue of *S. cerevisiae* STL1, which has been extensively studied as a glycerol uptake protein. To determine whether KMAR_60179 also transported glycerol, the ∆PT strain overexpressing *KMAR_60179*, was grown on medium with glycerol as the sole carbon source (Fig. 4). The growth impairment of *K. marxianus* ΔPT was rescued by KMAR_60179, confirming it as a glycerol, as well as a xylose, transporter (Fig. 5A). *Sc_STL1* and *KMAR_30579* (present in the same branch with Sc_STL1 and KMAR_60179 in Fig. 1) were overexpressed in the platform strain as well, to check their ability to rescue growth on both glycerol and xylose (Fig. 4). Any growth on glycerol conferred by these two transporters was minimal and they do not allow growth on xylose at either of the tested concentrations (2% and 0.1%). *In silico* modelling and comparison of the binding pockets of KMAR_60179 and Sc_STL1 offers a possible explanation for the difference in the capacity to transport xylose, since the binding pocket of Sc_STL1 appears tighter and may be unable to accommodate xylose (Fig. S7, Supporting Information). We cannot discount the possibility that *Sc_STL1* was not correctly expressed or localised but as the focus of this study was on *K. marxianus* genes, we did not investigate this further. To demonstrate the potential of the platform strain for the identification of heterologous transporters, *S. cerevisiae GAL2* was expressed in the ΔPT strain and assessed in the same manner as was done with the *K. marxianus* genes (Fig. 3; Table 3). As expected, GAL2 rescued the growth of the platform strain only on 2% xylose and arabinose 2% (plates) and allowed consumption of 31%–37% sugar in the liquid assay, confirming that, in its native form, it is a low affinity pentose transporter (Fig. 3) (Farwick *et al*. 2014).

**Table 3.** Growth and sugar consumption in 2% liquid medium. Each strain expressing the indicated gene was grown for 96 h with either 2% xylose or 2% arabinose as the sole carbon source. Biomass was determined by harvesting the cells and measuring dry cell weight (DCW) and sugar consumption was assessed by measuring residual sugar using HPLC. Dara were compiled from triplicate assays.

	Xylose 2%		Arabinose 2%	
	DCW (g L^−1^)	Sugar consumption (%)	DCW (g L^−1^)	Sugar consumption (%)
ΔPT	0.1	2.5 ± 2	0.1	0.98 ± 0.9
ΔPT + *KMAR_60179*	3.56 ± 0.04	52.64 ± 0.52	0.1 ± 0.2	0.4 ± 0.92
ΔPT + *KMAR_10527*	6.83 ± 0.33	90.42 ± 0.2	4.05 ± 0.63	45.36 ± 4.74
ΔPT + *KMAR_10528*	0.16 ± 0.05	3.23 ± 2.64	0.11 ± 0.015	2.46 ± 2.35
ΔPT + *KMAR_10529*	5.12 ± 0.40	66.28 ± 2.82	3.98 ± 0.48	42.16 ± 1.56
ΔPT + *KMAR_10530*	0.13 ± 0.005	4.73 ± 1.48	0.11 ± 0.005	7.47 ± 2.95
ΔPT + *KMAR_10531*	7.52 ± 0.34	94.63 ± 0.14	6.18 ± 0.5	65.85 ± 1.4
ΔPT + *KMAR_50027*	4.23 ± 0.05	59.34 ± 2.02	3.15 ± 1.41	59.5 ± 2.52
ΔPT + *KMAR_50342*	6.96 ± 0.36	79.93 ± 3.1	-	-
ΔPT + *KMAR_50343*	7.94 ± 0.82	85.1 ± 1.01	-	-
ΔPT + *KMAR_50344*	5.97 ± 0.61	55.58 ± 10.9	-	-
ΔPT + *KMAR_50345*	4.32 ± 0.40	58.29 ± 6.12	-	-
ΔPT + *KMAR_50346*	6.39 ± 2.15	68.61 ± 15.4	-	-
ΔPT + *Sc_GAL2*	2.73 ± 0.57	31.27 ± 1.8	2.03 ± 1.02	36.75 ± 1.25

### Xylose uptake is the main limiting factor for the complete consumption of this sugar in ∆PT strain

To assess the potential of *K. marxianus* ∆PT as a platform for functional characterization as well as identification of pentose transporters, *K. marxianus* ∆PT overexpressing either the high affinity transporter, KMAR_10531, or the low affinity transporter, KMAR_60179, was grown in liquid culture with sugar consumption monitored (Fig. 5). Following pre-growth on YPLAC, strains were inoculated in minimal medium with 2% xylose or 2% arabinose as the sole carbon source. Strains expressing either transporter displayed growth on xylose (Fig. 5A) but only KMAR_10531 supported growth on arabinose. Although the growth rate on xylose was not dramatically different, with slightly faster growth permitted by KMAR_10531 (μ _max_ of 0.06 h^−1 ^vs 0.07 h^−1^), there was an approximately two-fold difference in biomass generated: A_600_ ∼25 (KMAR_10531) *versus* A_600_ ∼13 (KMAR_60179). The strain expressing *KMAR_10531* also completely depleted xylose after 85h of growth whereas 50% of the xylose remained unused by the strain expressing *KMAR_60179* (Fig. 5A). On arabinose (Fig. 5B), the growth rate (μ_max_ 0.065 h^−1^) of the strain expressing *KMAR_10531* and the final cell density reached (A_600_ ∼23) were comparable to those on xylose. These findings are consistent with the data shown in Fig. 3 that indicated that KMAR_10531 is a high affinity transporter for both xylose and arabinose whereas KMAR_60179 is a low affinity xylose transporter and does not transport arabinose.

## DISCUSSION

The aims of this study were to expand our knowledge of sugar transporters in *K. marxianus* and to create a platform resource for study of pentose transporters in a native pentose-utilising yeast. This type of platform has been extensively used in *S. cerevisiae* for the functional study of native and heterologous sugar transporters, notably with strain EBY.VW4000, which lacks all functional hexose transporters (Alves-Araújo et al 2005; Pacheco *et al*. 2020; Wieczorke *et al*. 1999). More recently CRISPR-SpCas9 was used to delete the 21 hexose transporters in a *S. cerevisiae* CEN.PK background, to create a more stable strain for future studies (Wijsman *et al*. 2019). We successfully constructed an analogous platform in *K. marxianus* for the study of pentose transporters. *K. marxianus* ΔPT only contains 3 genomic deletions but lacks twelve genes. It is stable, grows well on lactose and fructose, and only requires the addition of a transport protein to grow on xylose or arabinose. This is desirable if metabolic or physiological studies are to be performed as manipulation of metabolic enzymes is not required. We illustrated the potential by confirming that two of the newly native *K. marxianus* identified transporters and *S. cerevisiae* GAL2 indeed displayed characteristics of pentose transport with high or low affinity. This platform can now be used for studies of transport kinetics, for characterisation of engineered transporters, and for identification of additional pentose transporters from other species.

One of the key questions that we wanted to address was how many of the *K. marxianus HGT*s encoded pentose transporters. We confirmed previous findings that KMAR_10527 (KmAxt1) is able to perform high affinity xylose/arabinose transport and determined that KMAR_10531 has similar capability. Two further HGTs, KMAR_10529 and KMAR_50027 are low affinity xylose/arabinose transporters. These four proteins appear to comprise the full capacity of *K. marxianus* to transport arabinose as deletion of the *HGT* cluster creates an arabinose-deficient strain. None of the HXT (KHT) proteins transported arabinose, though four were capable of low affinity xylose transport. This may well reflect a similar situation in *S. cerevisiae*, where only low affinity xylose transport occurs as that species lacks the *HGT* orthologues. Although the predicted low affinities might suggest that the *K. marxianus* HXT proteins are less significant than the high affinity HGT transporters for growth on xylose, it should be noted that it was necessary to delete the genes to create a xylose-negative platform strain (*K. marxianus* ΔPT), indicating that one or more of the HXTs is physiologically relevant. There is a conserved motif in the first transmembrane domain of MFS sugar transporters that is reported to be involved in substrate selectivity and kinetics so we compared this motif between the HGT and the HXT proteins (Knoshaug *et al*. 2015; Pacheco *et al*. 2020). All the HXT proteins have the glucose-specific motif sequence G-G-F-V-F-G-W-D, whereas the HGT proteins all have the motif X-G-X-X-F-G-F-D, as well as other similarities to known arabinose transporters (Fig. S7, Supporting Information). Another interesting feature is that three of the four HGT proteins that can transport arabinose were previously shown to be also able to transport galactose (Varela et al 2019a). These findings are consistent with a report that galactose transporters are often able to facilitate L-arabinose, due to the similar stereochemistry between those two sugars (Young *et al*. 2014). The correlation between galactose and arabinose transport was not absolute, however.

One of the more unexpected findings was that KMAR_60179, the *K. marxianus* equivalent of *S. cerevisiae* STL1, is a low affinity xylose transporter as well as a glycerol transporter. Sc_STL1 has been extensively characterised as a glycerol/H + symporter involved in the response to high osmolarity and there are no previous reports of its involvement in xylose transport (Ferreira and Lucas 2007; Ferreira *et al*. 2005; Zhao *et al*. 1994). Differences in the size of the binding pocket, which appears tighter than the one of KMAR_60179, could underlie this phenotype. Our preliminary work indicated that Sc_STL1 may not transport xylose but more detailed analysis to check expression, localisation, and other aspects, would be required to fully investigate this. Further work would also be needed to investigate whether STL1 plays the same role with respect to the osmotic response in both *K. marxianus* and *S. cerevisiae*.

The findings of this study are relevant both for fundamental understanding of sugar transport in yeasts and for biotechnological applications. For the latter, the new pentose transporters that we identified can serve as the basis for protein engineering for transporters with new kinetic functionality. In this respect, the fact that the best candidate transporters from *K. marxianus* are only distantly related to the xylose-transporting HXT derivative (GAL2) is positive as it indicates that the solution space for pentose transporters is quite large. There are also implications for the study of sugar transport and the evolution of transport proteins, though the study also raises as yet unanswered questions in this area. In general, it is difficult to conclude that just because a protein can transport a sugar when expressed heterologously, it does this under normal conditions. Nonetheless, the results with the deletions suggest that most of the proteins with which we worked make some contribution to transport of the relevant sugars. It also seems likely, however, that at least some of the HGTs, and indeed the other MFS transporters identified, transport as yet unknown substrates. It is not known why *K. marxianus* displays this relatively recent expansion of *HGT* genes but it is clear that the proteins are evolving to be functionally distinct. Further study of these proteins may offer new insights into some of the forces and processes that underlie yeast evolution.

## Supplementary Material

foab026_Supplemental_File
