# Supplementary material for: Identification of novel pentose transporters in Kluyveromyces marxianus using a new screening platform
Source: FEMS Yeast Res. 2021 Apr 23;21(4):foab026. doi: 10.1093/femsyr/foab026 (PMC8110514; doi:10.1093/femsyr/foab026)
Supplement: foab026_Supplemental_File [file foab026_supplemental_file.docx]

**Supplementary material Tables**

**Supplementary Table 1**. Equivalence of KHT and HGT genes in different *K. marxianus* genomes. Genes marked with an asterisk have arisen by recombination between two genes. / indicates that the gene is absent.

| **Homology** | ***NBRC1777*** | ***CBS 6556*** | ***DMKU3-1042*** |
| --- | --- | --- | --- |
|  |  |  |  |
| KHT-like | *KMAR_50347* | *KMXK_0E03690* | *KLMA_50364* |
|  |  |  |  |
| KHT-like | *KMAR_50346* | *KMXK_0E03680* | *KLMA_50363* |
|  |  |  |  |
| KHT-like | *KMAR_50345* | *KMXK_0E03670* | *KLMA_50362* |
|  |  |  |  |
| KHT-like | *KMAR_50344* |  |  |
|  |  | *KMXK_0E03660** | *KLMA_50361** |
| KHT-like | *KMAR_50343* |  |  |
|  |  |  |  |
| KHT-like | *KMAR_50342* | *KMXK_0E03650* | *KLMA_50360* |
|  |  |  |  |
|  |  |  |  |
| HGT-like | *KMAR_10527* | *KMXK_0A02960* | *KLMA_10545* |
|  |  |  |  |
| HGT-like | *KMAR_10528* | *KMXK_0A02950* | *KLMA_10546** |
|  |  |  |  |
| HGT-like | *KMAR_10529* | *KMXK_0A02940* | */* |
|  |  |  |  |
| HGT-like | *KMAR_10530* | *KMXK_0A02930* | *KLMA_10546** |
|  |  |  |  |
| HGT-like | *KMAR_10531* | *KMXK_0A02920* | *KLMA_10547* |
|  |  |  |  |
|  |  |  |  |
| LAC12-3p | *KMAR_20788* | *KMXK_0B08450* | *KLMA_20830* |
|  |  |  |  |
| LAC12-1p | *KMAR_30003* | *KMXK_0H00120* | *KLMA_30010* |
|  |  |  |  |
| LAC12-2p | *KMAR_30701* | *KMXK_0C07340* | *KLMA_30728* |
|  |  |  |  |
| LAC12-4p | *KMAR_80005* |  | *KLMA_80005* |
|  |  |  |  |
|  |  |  |  |
| YDR387C | *KMAR_20551* |  | *KLMA_20580* |
|  |  |  |  |
| YBR241C | *KMAR_20383* |  | *KLMA_20402* |
|  |  |  |  |
| Polyol transporter 2 | *KMAR_20602* |  | *KLMA_20638* |
|  |  |  |  |
| STL1 | *KMAR_30579* |  | *KLMA_30607* |
|  |  |  |  |
| HGT-like | *KMAR_50027* | *KMXK_E00380* | *KLMA_50032* |
|  |  |  |  |
| STL1 | *KMAR_60179* | *KMXK_0F03890* | *KLMA_60180* |
|  |  |  |  |
| Glucose sensor | *KMAR_60499* |  | *KLMA_60507* |
|  |  |  |  |
| YFL040W | *KMAR_70042* |  | *KLMA_70050* |
|  |  |  |  |
|  | *KMAR_70126* |  | *KLMA_70145* |
|  |  |  |  |
| FTR1 | *KMAR_80099* |  | *KLMA_60440* |
|  |  |  |  |
| ITR1/2 | *KMAR_80266* | *KMXK_0G02840* | *KLMA_80273* |
|  |  |  |  |
| Hxt14 | *KMAR_80370* | *KMXK_0G03930* | *KLMA_80389* |
|  |  |  |  |
|  | *KMAR_80407* |  | *KLMA_70003* |

**[Supplementary Table 2.](file:///C:\\Users\\loren\\il%20tuo%20team%20Dropbox\\Lorena%20Donzella\\New%20folder\\YEASTDOC\\UCC\\paper\\paper's%20versions\\02-12-20%20final%20draft%20Lorena_JM.docx#SupTabl1)** Primers used in this study for CRISPR gRNA target design, CRISPR-CAS9 genomic indel mutations diagnosis and *KMAR_60179* repair fragment construction.

The gRNA primers are designed to be 20nt in length and not include the PAM sequence at the 3’ end (NGG). For cloning into the CRISPR-plasmid pUCC001, the forward gRNA target primers present overhangs 5’-cgtc-3’ and the reverse target primers 5’-aaac-3’ (indicated in lower case).

For clean disruption of *KMAR_60179*, the repair fragment was constructed using primers RF_60179 F and R of 95 bp which overlap for 15 bp (indicated with capital letters).

| **gRNA Targets CRISPR** | |  |
| --- | --- | --- |
| *KMAR_20551*_Target_1F | cgtcCGGTAGGAAGTATACTTTGA |  |
| *KMAR_20551*_Target_1R | aaacTCAAAGTATACTTCCTACCG |  |
| *KMAR_50027*_Target_1F | cgtcGTGATTGCAGGTATGGGTGT |  |
| *KMAR_50027*_Target_1R | aaacACACCCATACCTGCAATCAC |  |
| *KMAR_30579*_Target_1F | cgtcTCAAACTGTCTCGTGGAGAA |  |
| *KMAR_30579*_Target_1R | aaacTTCTCCACGAGACAGTTTGA |  |
| *KMAR_60179*_Target_1F | cgtcACACTTCTACGATCCCAGTT |  |
| *KMAR_60179*_Target_1R | aaacAACTGGGATCGTAGAAGTGT |  |
| *KMAR_all_KHT*_Target 1F | cgtcTAGTTGGTACAGTAACCCAA |  |
| *KMAR_all_KHT*_Target_1R | aaacTTGGGTTACTGTACCAACTA |  |
| **Diagnostic primers** | |  |
| *KMAR_50347*_diag-F | ATGTCTAATCAATTGACGGC |  |
| *KMAR_50342*_diag-R | TTATTTCATCATAGCCTTGTACCATG |  |
| *KMAR_20551*_diag-F | GTACTCCACTGTTTCACAGCT |  |
| *KMAR_20551*_diag-R | CTCCATGCTGCCGGA |  |
| *KMAR_50027*_diag-F | TACTTCAATCACCCGAATGCA |  |
| *KMAR_50027*_diag-R | CTGCGCATCAATTATCACTTG |  |
| *KMAR_30579*_diag-F | CCTTTTGACCAACAAAGAATCC |  |
| *KMAR_30579*_diag-R | CCCCATAACACATTGAATAAACAATG |  |
| *KMAR_60179*_diag-F | AGCAATGACAGACACGC |  |
| *KMAR_60179*_diag-R | ACGGTTGACAGCGTC |  |
| ***KMAR*_60179 Repair fragment** | | |
| RF_60179_F | ggtcggacggtctttgagaatcgccatcacggtggtggcggttaccggtttttcgctattcgggtacgaccaaggtttgaTGTCCGGTATTATTAAACCCACAGGCTGAA | |
| RF_60179_R | ttttcagcggtacccgagacgtcttcttcttcttctggggaagtttgggagtctacaggagcagcagcagcagcatcagcTTCAGCCTGTGGGTTTAATAATACCGGACA | |

**Supplementary Table 3**. Primers used for the construction of plasmids overexpressing candidate transporters and to check the correct assembling. The Sc terminator CYC1 was amplified from S. cerevisiae CEN.PK with primers Sc_CYC1-F and R and cloned into a vector plasmid YTK001. The used primers present overhangs to allow the easy assembling by Golden Gate in the backbone TUDO-G418. The primers used to amplify and clone candidate transporters from scratch are designed to have overhangs containing 4 bp sticky ends to directionally clone the gene between the Sc_TEF1 promoter (in green) and Sc_CYC1 terminator (in red).

Primers ScTEF1-F and ScCYC1-R were used to amplify the cassette promoter- HGT/KHT-terminator presented in plasmid p426 under control of ScTEF1 promoter and ScCYC1 terminator.

Primers KIF and BsmbI_tudog418_R, annealing on the connectors flanking the expression cassette, were used to check the correct assembly of the TUDO-G418 plasmid with the coding sequence by colony PCR and sequencing.

| **Overexpression HGTs, KHTs and *KMAR_60179*, *KMAR_30579* and Sc_*STL1*** | |
| --- | --- |
| ScCYC1-F | gcatcgtctcatcggtctcaatccTCATGTAATTAGTTATGTCACGCTTACATTC |
| ScCYC1-R | atgccgtctcaggtctcacagcGCAAATTAAAGCCTTCGAGCGT |
| YTK_60179_F | gcatcgtctcatcggtctcatatgATGCTAAACAAAGTATCTGC |
| YTK_60179_R | atgccgtctcaggtctcaggatTTATTCCGAGCTGTTGGATG |
| YTK_10529_F | gcatcgtctcatcggtctcatatgATGTCATTTAAAGACAAGTTTTC |
| YTK_10529_R | atgccgtctcaggtctcaggatTTAAACGCTGTTACCAGAG |
| YTK_50346_F | gcatcgtctcatcggtctcatatgTTAGTTCTTCTTGAAGGACATGCG |
| YTK_50346_R | atgccgtctcaggtctcaggatATGTCCAATCAATTAACGGATACTACC |
| YTK_50347_F | gcatcgtctcatcggtctcatatgTTATTTCAAAGAAATTCTCTTATACCATGGCT |
| YTK_50347_R | atgccgtctcaggtctcaggatATGTCTAATCAATTGACGGCAAGT |
| YTK_50027_F | gcatcgtctcatcggtctcatatgATGACATTGCTGGACAAGC |
| YTK_50027_R | atgccgtctcaggtctcaggatTTAATTAGATTGACTGTTGGAATCAGT |
| YTK_30579­_F | gcatcgtctcatcggtctcatatgTCAGATTCTAAGAAAAGCG |
| YTK_30579­_R | atgccgtctcaggtctcaggatTTAGGAGGAAGGTGTTTCATCA |
| YTK_Sc_STL1­_F | gcatcgtctcatcggtctcatatgAAGGATTTAAAATTATCGAATTTCAAAG |
| YTK_Sc_STL1­_R | atgccgtctcaggtctcaggatTCAACCCTCAAAATTTGCTTTAT |
| YTK_Sc_GAL2­_F | gcatcgtctcatcggtctcatatgGCAGTTGAGGAGAACAATAT |
| YTK_Sc_GAL2­_R | atgccgtctcaggtctcaggatTTATTCTAGCATGGCCTTGTA |

**Primers to the check of the correct cloning assembly in the overexpression plasmids**

| K1F | tttgctggccttttgctc |
| --- | --- |
| BsmbI_tudog418_R | CCCCGTCGTCTCATTGGgcaaattaaagccttcgagc |
| ScTEF1-F | gcccaatacgcaaaccgcctctcccCATAGCTTCAAAATGTTTCTACTC |
| 50342-F | aatctaatctaagATGTCCGAAGCTGCTGGTTTAC |
| 50342-R | actaattacatgaTTATTTCATCATAGCCTTGTACCATG |
| 50343-F | gcatagcaatctaatctaagATGTCTGAAGCTGCTGCTGATTTACA |
| 50343-R | tgacataactaattacatgATTAATGCTTCATCATGGCCTTGTACCA |
| 50344-F | gcatagcaatctaatctaagATGTCTGAAGCTGCCGCTGA |
| 50344-R | gcgtgacataactaattacatgaCTCGAGGTCGACTTAATGCTTCAACAAAGCCTTGTACCAT |
| 50345_F | gcatagcaatctaatctaagATGTCTGAAGAAGCTGCATTACAG |
| 50345_R | tgacataactaattacatgaTTAGGACGTCATGCGCTTG |
| 10531_F | gcatagcaatctaatctaagATGACTTTAAAAGATAAACTATTGCTCC |
| 10531_R | tgacataactaattacatgaCTAGACCGAGCTGCTGCTATTAG |
| 10530_F | gcatagcaatctaatctaagATGTCATTCTTAGACAAGAAAAC |
| 10530_R | tgacataactaattacatgaTCAAACATTGTCATTCCTTAC |
| 10528_F | gcatagcaatctaatctaagATGAAACAATTCGCTACG |
| 10528_R | tgacataactaattacatgaTTATACGCGAGAGTCGTTC |
| 10527_F | gcatagcaatctaatctaagATGTCATTGAAAGACAAGATTTTG |
| 10527_R | tgacataactaattacatgaTTAGTTAGAGTTTGAGTTTGAGTTG |

**Supplementary Figures**

**S1.** **Construction of KHT deletion mutants on *K. marxianus* NBRC1777 wild type and Δhgt background.** Scheme showing the repair event occurred upon targeting a common sequence (in grey) of the KHT genes with the CRISPR-Cas9 system. Recombination between the two ends of the KHT loci is shown by grey shading. Recombination event between *KMAR_50347* and *KMAR_50342* led to the loss of functionality of the KHT cluster. The recombined sequence of 1685 bp contains: 490 bp of *KMAR_50347*, 80 bp of *KMAR_50346*, 72 bp of *KMAR_50343* and 1043 pb of *KMAR_50342*. The stop codon (marked with a red asterisk in position 721-723), obtained as consequence of the deletion, results in two truncated proteins of 240 AA and 321 AA, respectively, both lacking 7 fundamental transmembrane domains to be functional. Primers *KMAR_50347*_diag_F and *KMAR_50342*_diag_R used to screen the mutant colonies are shown as red arrows.

**
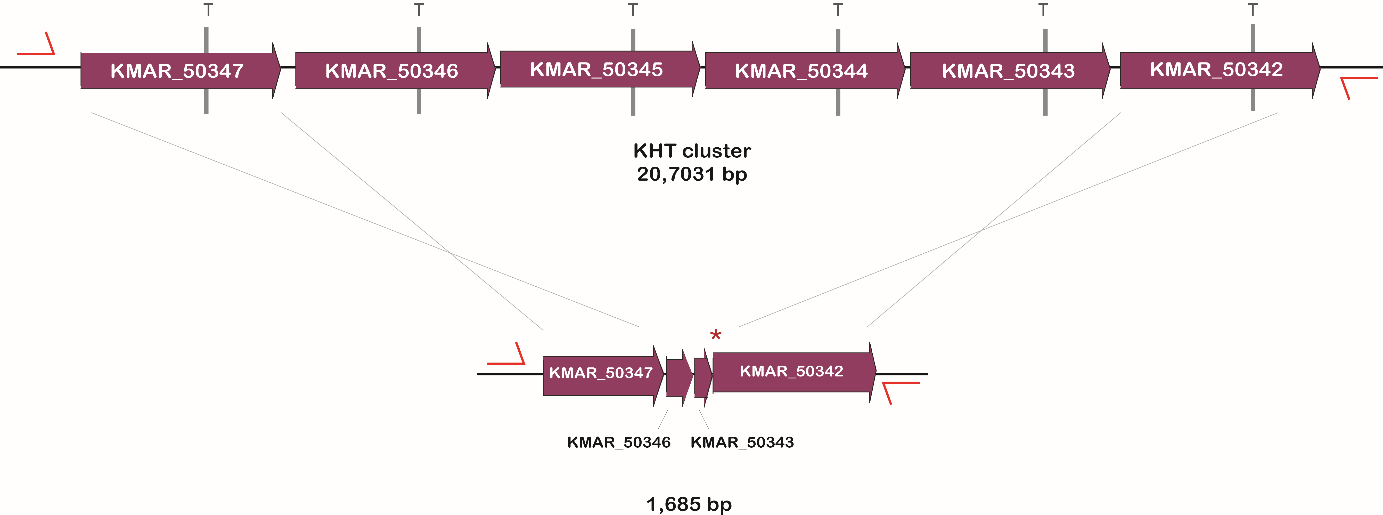
**

**S2. Schematic representation of *KMAR_60179* clean deletion**. For disruption of *KMAR_60179*, a repair fragment was constructed using *K. marxianus* NBRC 1777 genomic DNA as the template, designing primers RF_60179 F and R for the homology arms at the extremities of *KMAR_60179* of 95 bp (in pink). The 5’ and 3’ homology arms were then joined by overlap extension PCR. The final repair fragment of 190bp was then co-transformed with the CRISPR-Cas9 plasmid pUCC001-60179 (carrying a specific target sequence recognized by the Cas9 complex, showed in grey). into *K. marxianus* ΔhgtΔkht to generate *K. marxianus* ΔhgtΔkhtΔKMAR_60179 (later named *K. marxianus* ΔPT). The deletion mutant was confirmed by diagnostic colony PCR and sequencing across the deletion with primers 60179_F and 60179_R.

**
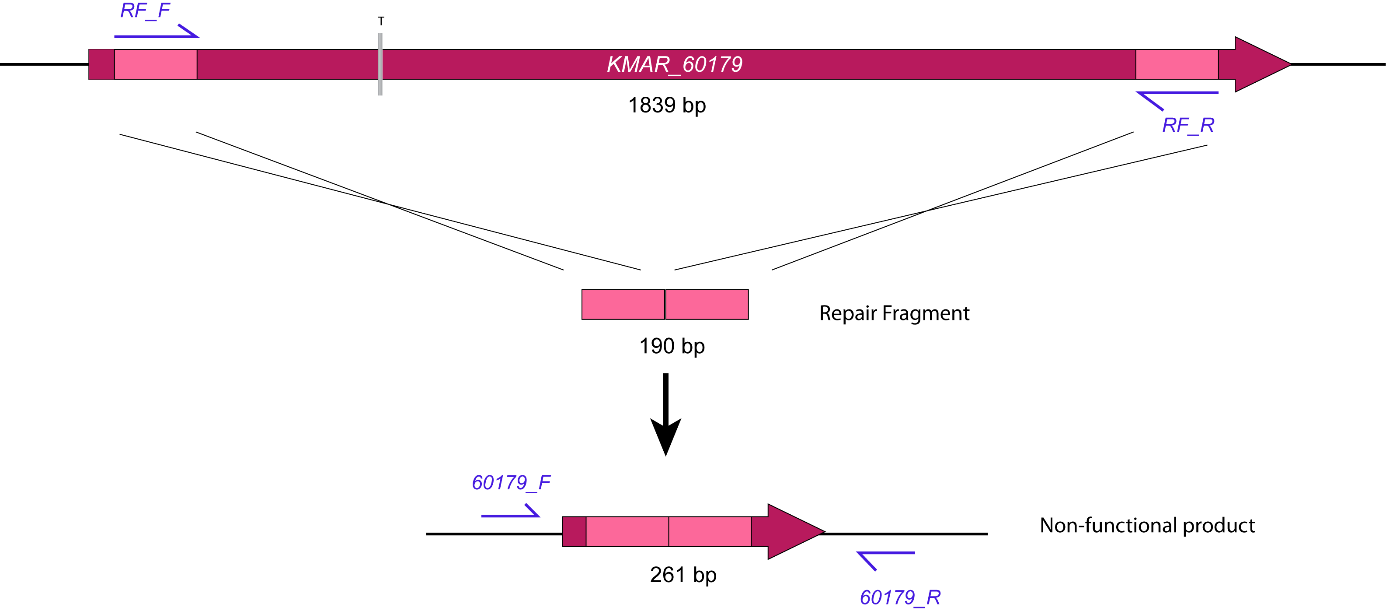
**

**S3**. **A scheme summarizing the complementation assay performed in order to overexpress interesting transporters in *K. marxianus* ∆PT.** ScTEF1 promoter, ScCYC1 terminator from *S. cerevisiae* and the relevant genes from *K. marxianus* were amplified by PCR with primers containing specified 4bp overlaps. PCR products were then purified and assembled in pTUDOG418 using a BsaI Golden Gate assembly. The resulting plasmids were then introduced in competent *E. coli* cells by heat shocked and plated on LB+ Amp. After 24 h, white colonies, in which the GFP was substitute with the expression cassette, were screened by colony PCR, plasmids were extracted and the platform strain *K. marxianus* NBRC1777 ΔPT was transformed with selection for G418. Spotted test with serial dilutions on MM plates with lactose, xylose, arabinose and glycerol to test the capacity of wt and engineered strains to use specific sugars.


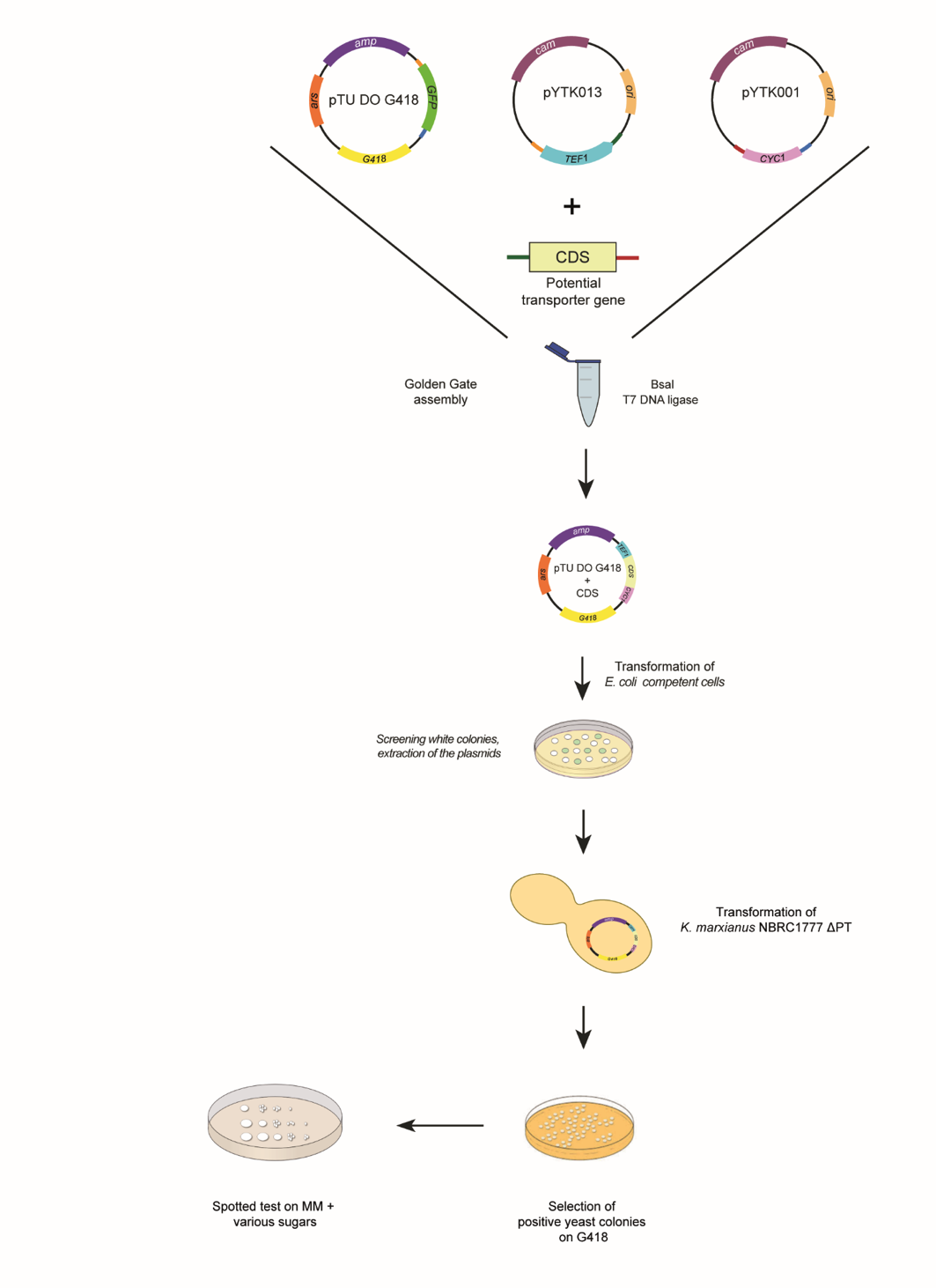


**S4.** **Maximum-likelihood phylogenetic analysis of sugar transporters in *K. marxianus* compared with known xylose-arabinose transporters Xut1 (XP_001385583) and Xut3 (XP_001387138) of *S. stipitis*, Xylh (XP_458169) of *D. hansenii*, Gal2 N376F (NP_013182) of *S. cerevisiae*, Axt1 (XP_002562738) of *P. guiliermondii* and AraT (XP_001482096)of *P. chrysogenum*** (in green). The candidate transporters knocked out in *K. marxianus* NBRC1777 to create the pentose transporters null strain ΔPT are reported in red.

*
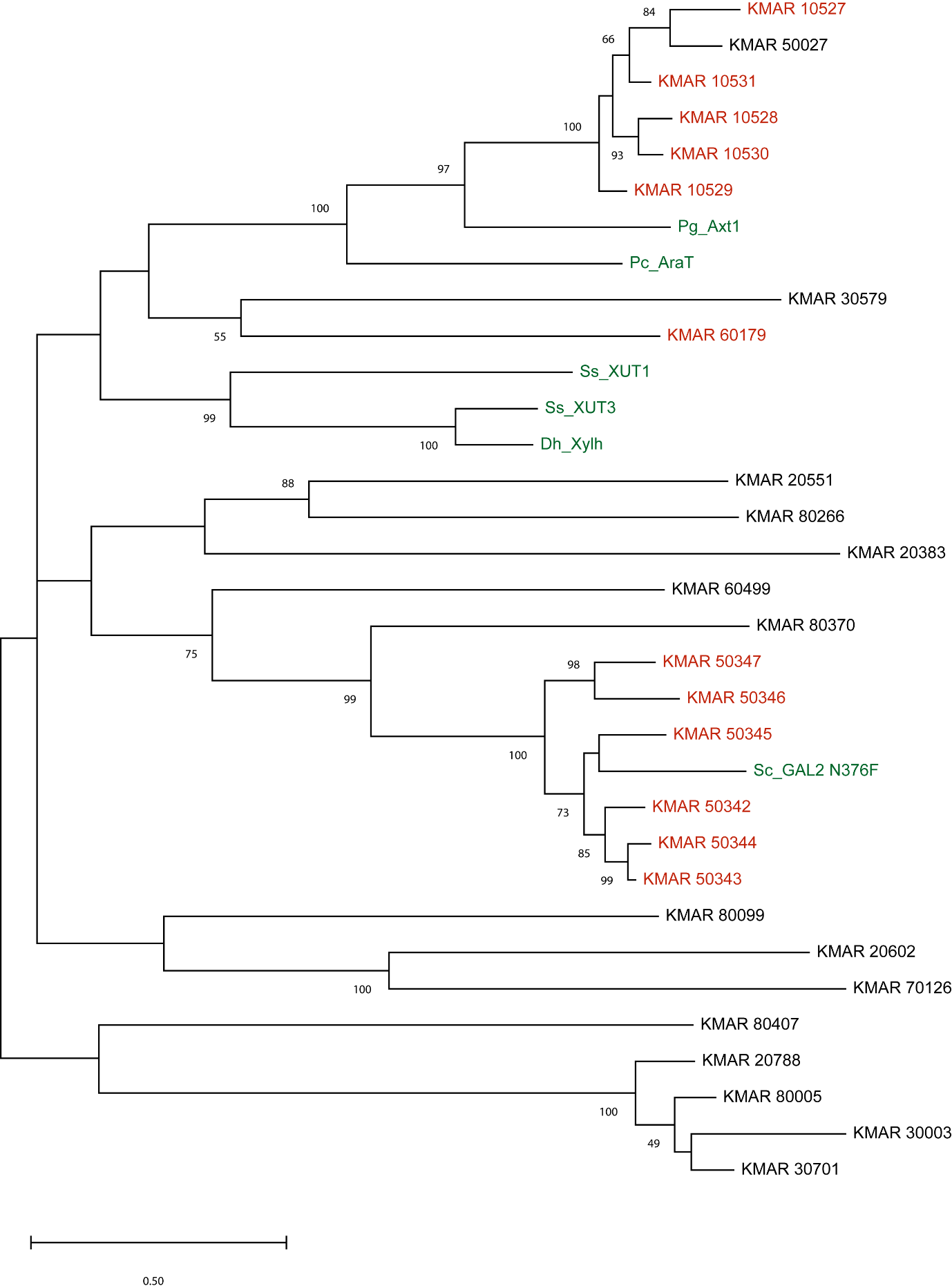
*

**S6. KMAR_60179 (on the left) and Sc_STL1 (on the right) binding sites**. Amino acids within 4 Å from the ligand (xylose in pink) are reported. The amino acids in the binding pockets of the two transporters result to be highly conserved and the main difference is in the size of the pore, being the one of Sc-STL1 tighter due to the presence of the ASN308 really close to the ligand.


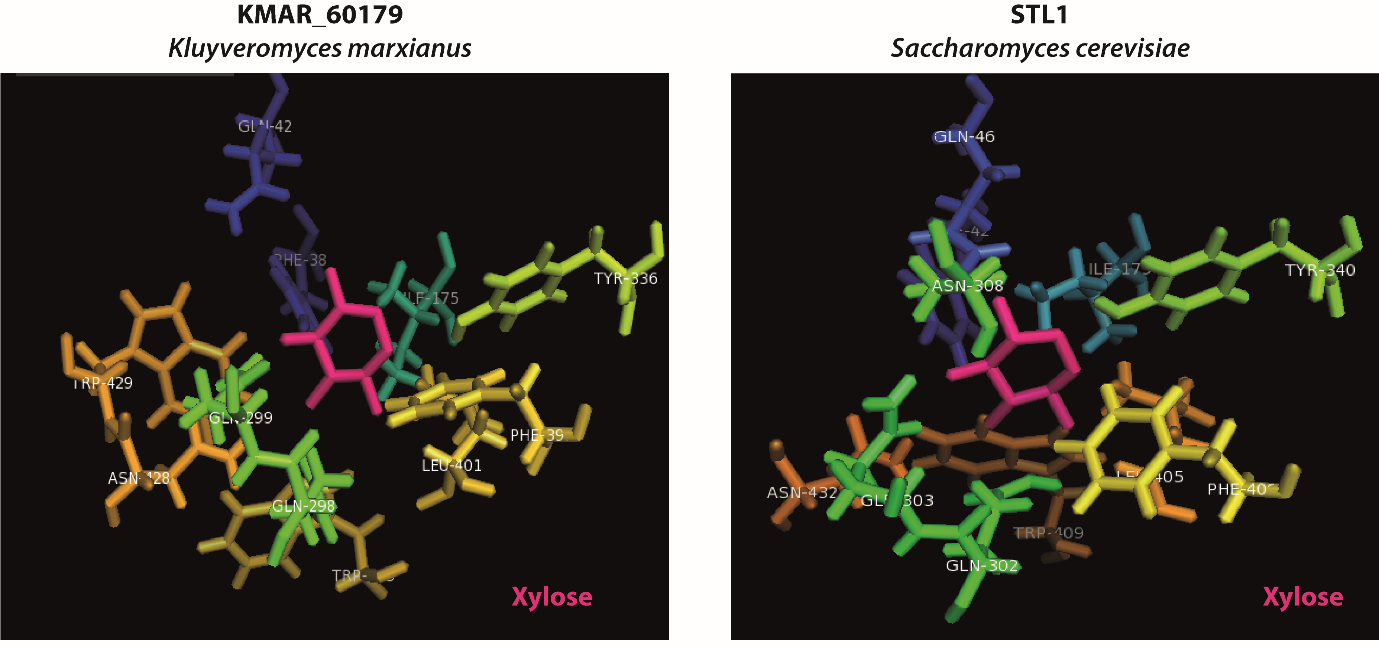


**S7.** **Comparison of a conserved motif in the first transmembrane span** among promising sugar transporters of *K. marxianus*, specific xylose transporters of *S. stipitis* (XUT1 and XUT 3) and *D. hansenii* (Xylh) and arabinose transporters of *P. chrysogenum* (AraT) and *P. guilliermondii* (Axt1).


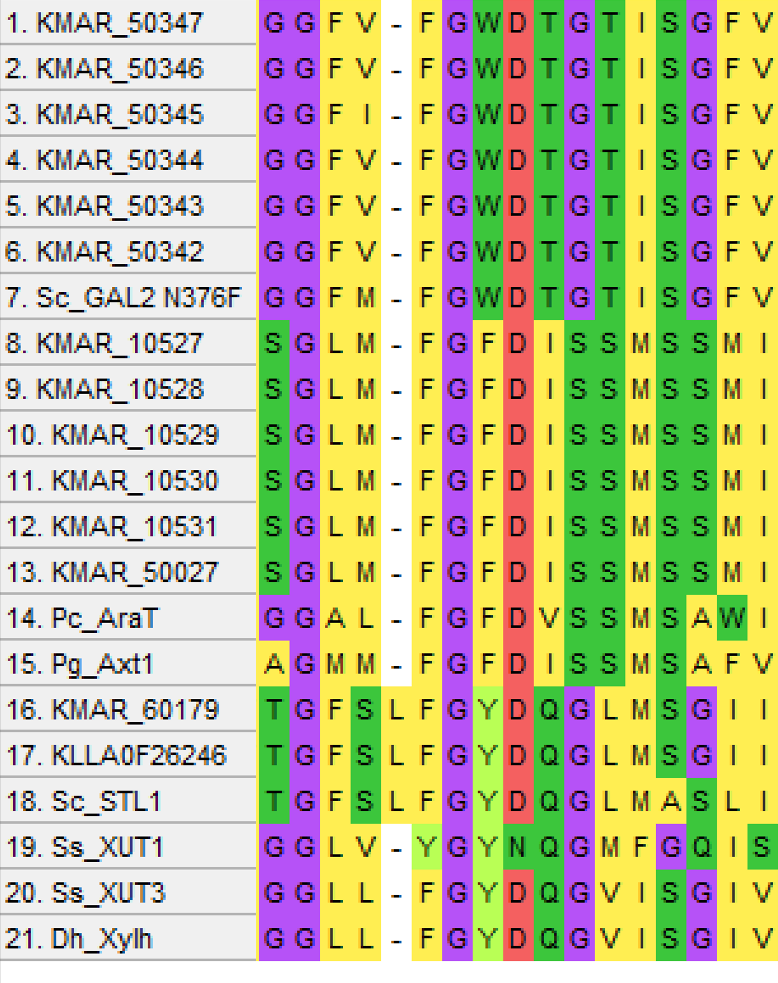


**S8.** **Growth phenotype of *K. marxianus* putative pentose transporters null strains.** Yeast strains were grown on YPLAC to an OD_600_ of 2 and then washed, serial diluted and spotted onto MM plates supplemented with lactose or xylose 2%. No major differences in growth between ∆PT and the other mutants were observed on YPLAC plates.


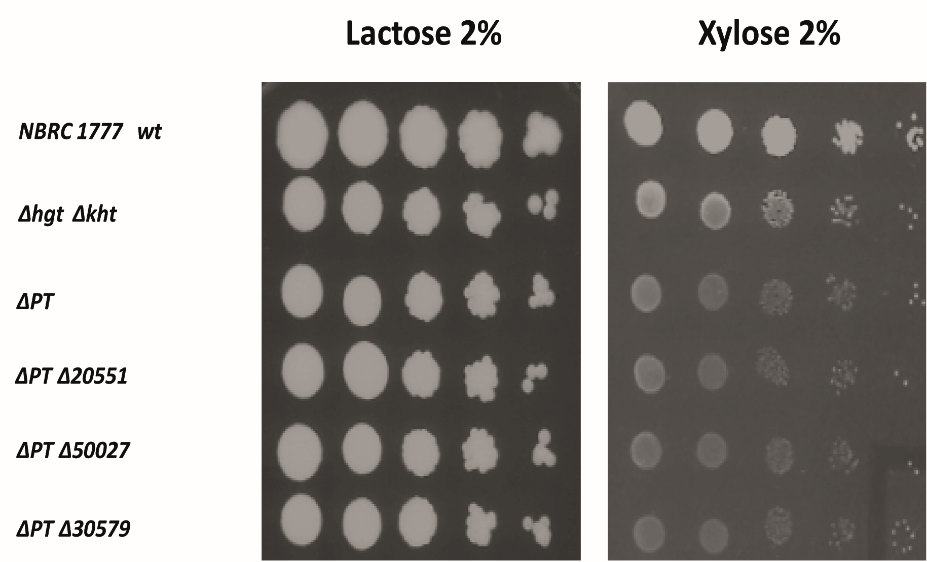


**Supplementary File 1**

**1000x vitamin solution for minimal medium**

1. Dissolve 50 mg of D(+) biotin in 10 mL 0.1 M NaOH (made in sterile water).

2. Add this to 750 mL of deionized water and adjust the pH to 6.5 with 1M HCl.

3. Add the following compounds and adjust the pH to 6.5 after each addition:

- D-calcium pantothenate 1 g
- Nicotinic acid 1 g
- Myo-inositol 25 g
- Thiamine hydrochloride 1 g
- Pyrixodine hydrochloride 1 g
- para-aminobenzoic acid 0.2 g

4. Wait for every compound to finish dissolving before adding the next

5. Adjust the pH to a final value of 6.5 and make up the volume to 1L.

6. Sterilize by filtration.

7. In a laminar flow bench, dispense the medium into aliquots and store at 4 °C.

**1000x trace element solution for minimal medium**

1. Dissolve the following in 750 mL of deionized water (adjust to pH 6):

- EDTA disodium salt dihydrate 15 g
- Zinc sulphate heptahydrate (ZnSO_4_.7H_2_O) 4.5 g

2. Add the following compounds in the given order and adjust the pH to 6.5 after each

addition:

- Manganese (II) chloride dihydrate (MnCl_2_.2H_2_O) 0.84 g
- Cobalt chloride hexahydrate (CoCl_2_.6H_2_O) 0.3 g
- Coppersulphate pentahydrate (CuSO_4_.5H_2_O) 0.3 g
- Sodium molybdate dihydrate (Na_2_MoO_4_.2H_2_O) 0.4 g
- Calcium chloride dihydrate (CaCl_2_.2H_2_O) 4.5 g
- Iron sulphate heptahydrate (FeSO_4_.7H_2_O) 3 g
- Boric acid (H_3_BO_3_) 1 g
- Potassium iodide (KI) 0.1 g

3. Wait for each salt to dissolve before adding the next one. If you do not do this or fail to

adjust the pH, everything will turn into a mossy green insoluble sludge.

4. At the end, adjust the pH to 4.0 and make up the volume to 1 L.

5. Sterilize by autoclaving and store at 4 °C.

6. The solution will initially be green but slowly turn purple over time.
